# Measuring respiratory symptoms of COPD: performance of the EXACT- Respiratory Symptoms Tool (E-RS) in three clinical trials

**DOI:** 10.1186/s12931-014-0124-z

**Published:** 2014-10-07

**Authors:** Nancy K Leidy, Lindsey T Murray, Brigitta U Monz, Linda Nelsen, Mitchell Goldman, Paul W Jones, Elizabeth J Dansie, Sanjay Sethi

**Affiliations:** Evidera, 7101 Wisconsin Ave, Suite 1400, Bethesda, MD 20814 USA; Formerly Boehringer Ingelheim GmbH, Ingelheim, Germany; Merck, Sharp & Dohme, Corp, Whitehouse Station, NJ USA; AstraZeneca, Wilmington, DE USA; St. George’s, University of London, London, UK; University of Buffalo, Buffalo, NY USA

**Keywords:** COPD, Respiratory symptoms, Clinical trials, Dyspnoea, Cough, Sputum, Chest symptoms

## Abstract

**Background:**

Symptomatic relief is an important treatment goal for patients with COPD. To date, no diary for evaluating respiratory symptoms in clinical trials has been developed and scientifically-validated according to FDA and EMA guidelines. The EXACT – Respiratory Symptoms (E-RS) scale is a patient-reported outcome (PRO) measure designed to address this need. The E-RS utilizes 11 respiratory symptom items from the existing and validated 14-item EXACT, which measures symptoms of exacerbation. The E-RS total score quantifies respiratory symptom severity, and 3 domains assess breathlessness, cough and sputum, and chest symptoms.

**Methods:**

This study examined the performance of the E-RS in each of 3 controlled trials with common and unique validation variables: one 6-month (N = 235, US) and two 3-month (N = 749; N = 597; international). Subjects completed the E-RS as part of a daily eDiary. Tests of reliability, validity, and responsiveness were conducted in each dataset.

**Results:**

In each study, RS-Total score was internally consistent (Cronbach α) (0.88, 0.92, 0.92) and reproducible (intra-class correlation) in stable patients (2 days apart: 0.91; 7 days apart: 0.71, 0.74). RS-Total scores correlated significantly with the following criterion variables (Spearman’s rho; p < 0.01, all comparisons listed here): FEV_1_% predicted (−0.19, −0.14, −0.15); St. George’s Respiratory Questionnaire (SGRQ) (0.65, 0.52, 0.51); Breathlessness, Cough, and Sputum Scale (BCSS) (0.89, 0.89); modified Medical Research Council dyspnoea scale (mMRC) (0.40); rescue medication use (0.43, 0.42); Functional Performance Inventory Short-Form (FPI-SF) (0.43); 6-minute walk distance (6-MWT) (−0.30, −0.14) and incremental shuttle walk (ISWT) (−0.18) tests. Correlations between these variables and RS-Breathlessness, RS-Cough and Sputum, RS-Chest Symptoms scores supported subscale validity. RS-Total, RS-Breathlessness, and RS-Chest Symptoms differentiated mMRC levels of breathlessness severity (p < 0.0001). RS-Total and domain scores differentiated subjects with no rescue medication use and 3 or more puffs (p < 0.0001). Sensitivity to changes in health status (SGRQ), symptoms (BCSS), and exercise capacity (6MWT, ISWT) were also shown and responder definitions using criterion- and distribution-based methods are proposed.

**Conclusions:**

Results suggest the E-RS is a reliable, valid, and responsive measure of respiratory symptoms of COPD suitable for use in natural history studies and clinical trials.

**Trial registration:**

MPEX: NCT00739648; AZ1: NCT00949975; AZ 2: NCT01023516

**Electronic supplementary material:**

The online version of this article (doi:10.1186/s12931-014-0124-z) contains supplementary material, which is available to authorized users.

## Background

Respiratory symptoms, including breathlessness, cough, and sputum production/expectoration, are defining features of chronic obstructive pulmonary disease (COPD) that can adversely affect patient functioning and quality of life [1-10]. Symptomatic relief is often the patient’s primary concern, an important treatment goal for clinicians, and a key outcome in clinical intervention trials. With no known cure for COPD, effective symptom palliation and exacerbation prevention are paramount.

The EXACT – Respiratory Symptoms (E-RS) scale was designed to serve as a primary, secondary, or exploratory endpoint in clinical trials evaluating the effect of treatment on respiratory symptoms of COPD. The E-RS is based on the 11 respiratory symptom items from the 14-item EXACT, a daily diary used to measure exacerbations of COPD [11-15] (see Additional file 1: Table E7). The E-RS yields a total score, quantifying respiratory symptom severity overall, and 3 subscale scores assessing breathlessness; cough and sputum; and chest symptoms. This permits 2 validated uses for a single diary: quantification of respiratory symptoms in stable COPD using E-RS total and subscale scores and the assessment of acute exacerbations (frequency, severity, duration of symptom-defined events, and change in exacerbation symptoms with medically-treated events) using the EXACT total score [15,16].

Good research practice and Food and Drug Administration (FDA) guidelines [17-19] for patient-reported outcome (PRO) measures were followed during E-RS development [15,17-19]. Qualitative research was performed to assess content validity and reliability, validity, and sensitivity to change were tested in data from a prospective naturalistic study in the United States (US) [20]. To date, performance properties of E-RS scores within the context of international randomized controlled trials (RCTs) had not been established.

The objective of this study was to test the performance of E-RS scores in each of 3 independent, international RCTs evaluating 2 experimental drugs for the treatment of COPD.

## Methods

### Study design, sample, and procedures

Pre-specified secondary analyses were performed on data from 3 Phase II multi-centre, randomized, double-blind, placebo-controlled trials. In each trial, patients were enrolled during a stable state and completed the 14-item EXACT daily diary as part of the trial procedures. These datasets were also used to test the EXACT for evaluating exacerbations of COPD that occurred during the course of the trials [14].

The first dataset (Mpex) was a 6-month trial conducted in the US testing MP-376 (Levofloxacin) Inhalation Solution administered for 5 days every 28 days to prevent exacerbations in high risk COPD patients (NCT00739648), with exacerbation rate over the study period serving as the primary efficacy endpoint. Relevant inclusion criteria were: age ≥40 years; post-bronchodilator forced expiratory volume in 1 second (FEV_1_) ≤70% predicted and FEV_1_/forced vital capacity (FVC) ≤0.7; history of 2 or more exacerbations the prior year; mucopurulent sputum on most days, even when exacerbation-free; and stable on long-acting bronchodilators and/or inhaled or systemic steroids during the 30 day pre-baseline period. Maintenance therapy for each patient was at the clinician’s discretion.

Two datasets were from 12-week, parallel-group, multi-national trials testing AZD9668 (a neutrophil elastase inhibitor). AstraZeneca (AZ) 1 was dose-ranging with patients on a baseline treatment of tiotropium (NCT00949975) [21]. AZ 2 tested 1 dose against placebo, with patients receiving budesonide/formoterol (NCT01023516) [22]. The primary efficacy endpoint for both trials was pre-bronchodilator FEV_1_. Relevant inclusion criteria were: age 40 to 80 years; post-bronchodilator FEV_1_% predicted 40%–80% (AZ 1) or 30%–80% (AZ 2); 1 or more clinic visit or hospitalization for exacerbation the prior year; Breathlessness, Cough, and Sputum Scale (BCSS) score ≥2 per day for at least 7 of 14 days before enrolment (Visit 2); and stable (no treatment, clinic visit or hospitalization for exacerbation) for at least 4 weeks prior to randomization.

Each study protocol stated that procedures adhered to the Declaration of Helsinki and institutional review boards/ethic committees approved the protocol(s) stipulating that all subjects would provide written informed consent prior to participation in the trial.

Due to differences in settings, maintenance therapies, and criterion (validation) variables, no cross-study pooling or analyses were planned or performed. Within each trial, the experimental drug showed no treatment effects on the primary or secondary endpoints. This allowed pooling of data across treatment groups within each database for the purpose of this psychometric validation, i.e., an examination of the performance properties of E-RS scores in 3 independent, international samples of stable patients with symptomatic, moderate to severe COPD undergoing treatment with maintenance therapies.

### Measures

All studies collected patient demographics, disease history, and clinical data, with variance across trials. The following assessments were relevant to these analyses.

#### Patient-reported measures

In each trial, participants completed an eDiary every evening prior to bedtime that included the E-RS, as part of the EXACT, and trial-specific assessments. Score ranges are shown in Table 1; higher scores indicate more severe symptoms. Participants in the Mpex trial also recorded daily global health ratings; those in the AZ trials completed the 3-item BCSS [23,24] and rescue medication use.

**Table 1:** **Sociodemographic and clinical characteristics**

	**Mpex**	**AZ 1**	**AZ 2**
	(N = 235)*	**(N = 749)** ^**†**^	**(N = 597)** ^**‡**^
Age, mean (SD)	63.7 (8.95)	62.3 (8.25)	61.7 (8.27)
Gender, n (% Male)	113 (48.1)	572 (76.4)	443 (74.2)
Race/Ethnicity, n (%)			
White	214 (91.1)	536 (71.6)	592 (99.2)
Asian	2 (0.9)	212 (28.3)	0 (0.0)
Black/African American	18 (7.7)	0 (0.0)	0 (0.0)
Hispanic or Latino	6 (2.6)	2 (0.3)	2 (0.3)
FEV_1_, mean (SD)^§^	1.2 (0.58)	1.7 (0.49)	1.6 (0.52)
FEV_1_% predicted, mean (SD)	42.2 (18.10)	58.8 (12.70)	54.2 (15.36)
GOLD^¶^ stage, n (%)			
0	22 (9.4)	0 (0.0)	0 (0.0)
I	2 (0.9)	23 (3.2)	22 (3.8)
II	55 (23.4)	503 (69.1)	300 (51.9)
III	89 (37.9)	197 (27.1)	240 (41.5)
IV	66 (28.1)	5 (0.7)	16 (2.8)
SGRQ total score^∥^	57.4 (16.21)	52.6 (18.36)	54.9 (17.14)
Exercise test meters, mean (SD)	289 (121)**	392 (122)**	337 (185)^††^
RS-Total Score, mean (SD)^‡‡^	15.7 (5.93)	15.9 (6.0)	18.2 (5.99)
RS-Breathlessness	8.2 (5.93)	7.9 (3.17)	8.9 (3.12)
RS-Cough and Sputum	4.1 (2.08)	4.2 (1.56)	4.7 (1.60)
RS-Chest Symptoms	3.3 (2.08)	3.8 (1.93)	4.6 (1.91)

^*^United States.

^†^Australia, Canada, Germany, Japan, Korea, Philippines, Poland, Russia, Slovakia, Taiwan, Ukraine, United States.

^‡^Bulgaria, Czech Republic, Hungary, Poland, Romania, Slovakia.

^§^Post-bronchodilator.

^¶^Spirometric classification GOLD 2014 [5].

^∥^Range: 0 to 100, higher scores = worse health status; SGRQ-C was used in both AZ studies.

**6 Minute Walk Test (6MWT).

^††^Incremental Shuttle Walk Test (ISWT).

^‡‡^Baseline, mean (SD) weekly scores, Day −7 to Day 1; Ranges: RS-Total: 0 to 40; RS-Breathlessness: 0 to 17; RS-Cough and Sputum: 0 to 11; RS-Chest Symptoms: 0 to 12; higher scores = more severe symptoms.

*Abbreviations:* *AZ* AstraZeneca, *FEV*_*1*_ forced expiratory volume in 1 second, *GOLD* Global Initiative for Chronic Obstructive Lung Disease, *SD* standard deviation, *SGRQ* St. George’s Respiratory Questionnaire, *SGRQ-C* St. George’s Respiratory Questionnaire for COPD.

During clinic visits, patients completed the St. George’s Respiratory Questionnaire (SGRQ) [25]. Those in the Mpex trial completed the Modified Medical Research Council (mMRC) scale [26], and patients in AZ 1 completed the Functional Performance Inventory – Short Form (FPI-SF), evaluating the ease or difficulty with which they perform daily activities across 6 domains [27,28].

#### Spirometry and exercise tolerance

At enrolment and subsequent clinic visits, spirometry and exercise tolerance tests (6-minute walk distance [6MWT] [Mpex and AZ 1] or incremental shuttle walk [ISWT] [AZ 2]) were performed.

### Analyses

A statistical analysis plan was developed for each dataset prior to analyses. Minimum data requirements were at least 4 days of baseline diary data (Day −7 to Day −1) and ≥80% of diary compliance for the period baseline to end of study or early termination date. Tests were performed on daily (Day −1) and mean weekly (Day −7 to Day −1) E-RS scores. Because results were consistent, results for mean weekly scores are reported unless otherwise specified. Analyses were performed with SAS/STAT software version 9.2 of the SAS System for PC (SAS Institute; Cary, North Carolina).

Internal consistency reliability of each E-RS scale was assessed using Cronbach’s alpha. Intraclass correlation coefficients (ICC) were used to evaluate reproducibility during trial run-in periods, Day −7 and Day −1, assuming symptomatic stability across these 2 observations. The daily global health assessments in the Mpex diary permitted 2-day test-retest analyses (Days 1–2) in the subset of patients reporting no change in lung condition. Paired *t*-tests and effect sizes (ES) were used to further understand E-RS score reproducibility.

Validity was assessed by examining the relationship between baseline E-RS scores and the following criteria, with variables determined by trial-specific data—airway obstruction: FEV_1_% predicted (all trials); respiratory symptom severity: BCSS (AZ 1, AZ 2); mMRC (Mpex); rescue medication use (AZ 1, AZ 2); health status: SGRQ (all trials); functional performance: FPI-SF (AZ 1); exercise capacity: 6MWT (Mpex, AZ 1) and ISWT (AZ 2). Spearman’s rho was used for analyses of correlation. Analysis of covariance (ANCOVA) was used to test E-RS score differences across mMRC classification; student’s *t*-test was used to test scores by rescue medication use at baseline (none versus ≥3 puffs per day averaged over 7 days).

Tests of responsiveness were conducted in sub-groups of patients experiencing improvements from baseline to 3 months using the following indicators and their respective responder definitions: health status (SGRQ ≥4 points) [29], symptoms (BCSS ≥1 point) [24], and exercise capacity (6MWT ≥26 meters or ISWT ≥47.5 meters) [30,31]. E-RS score changes were expressed in terms of mean (SD) and magnitude (percent and ES). Exploratory analyses examining E-RS score changes in subjects experiencing health status deterioration (SGRQ and BCSS) over 12 weeks were also performed.

Criterion-based values were examined in conjunction with distribution-based estimates (1/2 standard deviation and standard error of measurement [SEM]) to yield responder definitions, i.e., threshold estimates for meaningful symptomatic improvement. Descriptive statistics were used to examine magnitude of symptomatic (E-RS) change in responders and non-responders (mean and percent change, ES) using the proposed threshold, with figures showing mean (SD) weekly change over 12 weeks by responder status. Threshold estimates were also examined in non-responders showing symptomatic decline over this period.

## Results

### Sample

Sample demographic and clinical characteristics by study are shown in Table 1. Of those randomized, 235 (78%) [Mpex], 749 (89%) [AZ 1], and 597 (97%) [AZ 2] met the minimum data requirements for analysis. eDiary compliance rates from baseline to final visit for the 3 analytical samples were 87%, 94%, and 97%, respectively.

### Reliability

Internal consistency levels (Cronbach’s alpha) and reproducibility (ICC) for RS-Total and subscales are shown in Table 2. Weekly internal consistency levels exceeded 0.90 for the RS-Total, RS-Breathlessness, and RS-Chest Symptoms scales. The RS-Cough and Sputum subscale exceeded 0.70 [32,33] in 2 of the 3 trials. Two-day ICC levels in patients reporting no change (Mpex data) were greater than 0.80. Reproducibility estimates for the 6-day pre-treatment interval exceeded the recommended 0.6 threshold [34] in 11 of the 12 tests. The one exception was the RS-Cough and Sputum score in the Mpex study (ICC = 0.58; mean difference [SD] =0 [1.68] p = 0.75; ES = −0.02) (see Additional file 1: Tables E1–E3).

**Table 2:** **Internal consistency and reliability**

**Reliability parameter**	**Mpex**		**AZ 1**		**AZ 2**	
	**(N = 235)**		**(N = 749)**		**(N = 597)**	
Internal consistency*	**Daily** ^**†**^	**Weekly** ^**‡**^	**Daily** ^**†**^	**Weekly** ^**‡**^	**Daily** ^**†**^	**Weekly** ^**‡**^
RS-Total Score	0.88	0.90	0.92	0.94	0.92	0.95
RS-Breathlessness	0.89	0.93	0.90	0.94	0.90	0.94
RS-Cough and Sputum	0.40	0.52	0.68	0.73	0.71	0.78
RS-Chest Symptoms	0.92	0.96	0.89	0.94	0.87	0.93
Reproducibility of daily scores^§^	**Day 1–2**	**Day −7 to −1**		**Day −7 to −1**		**Day −7 to −1**
	**(N = 170)**	**(N = 235)**		**(N = 715)**		**(N = 597)**
RS-Total Score	0.91	0.71	N/A	0.74	N/A	0.74
RS-Breathlessness	0.87	0.73	N/A	0.71	N/A	0.74
RS-Cough and Sputum	0.84	0.58	N/A	0.71	N/A	0.69
RS-Chest Symptoms	0.89	0.69	N/A	0.71	N/A	0.69

*Cronbach’s alpha.

^†^Day −1.

^‡^Day −7 to Day −1.

^§^ICC, random-effects model, Day −7 to Day −1.

*Abbreviations:* *AZ* AstraZeneca, *ICC* intraclass correlation coefficient, *N/A* not available (no global assessment of day-to-day stability).

### Validity

Results of tests of construct validity are shown in Tables 3 and 4. E-RS scores were significantly correlated with indicators of airway obstruction, respiratory symptom severity, rescue medication use, health status, functional performance, and exercise tolerance. Among subscales, FEV_1_% predicted, mMRC, rescue medication use, exercise capacity, and functional performance were most strongly related to RS-Breathlessness.

**Table 3:** **Correlations** ^**†**^ **between E-RS scores and airway obstruction, respiratory symptom severity, and rescue medication use**

	**Mpex**		**AZ 1**			**AZ 2**		
	**FEV**_**1**_ **% predicted** ^**‡**^ **(N = 234)**	**mMRC** ^**‡**^ **(N = 235)**	**FEV**_**1**_ **% predicted** ^**‡**^ **(N = 746)**	**BCSS total score** ^**§**^ **(N = 735)**	**Rescue medication use** ^**§**^ **(N = 735)**	**FEV**_**1**_ **% predicted** ^**‡**^ **(N = 596)**	**BCSS total score** ^**§**^ **(N = 586)**	**Rescue medication use** ^**§**^ **(N = 597)**
E-RS Scores								
RS-Total Score	−0.19**	0.40***	−0.14***	0.89***	0.43***	−0.15***	0.89***	0.42***
RS-Breathlessness	−0.32***	0.46***	−0.17***	0.80***	0.42***	−0.21***	0.77***	0.43***
RS-Cough and Sputum	−0.05	0.16*	−0.12***	0.89***	0.38***	−0.06	0.92***	0.33***
RS-Chest Symptoms	0.03	0.27***	−0.07*	0.75***	0.33***	−0.09*	0.79***	0.36***

^†^Spearman's rank-order correlation; E-RS mean weekly scores at baseline (Day −7 to Day −1).

^‡^Clinic visit, Day 1, prior to treatment.

^**§**^Diary: BCSS scores at baseline (Day −7 to Day −1); rescue medication (baseline, Day −1).

**Bold** coefficients identify constructs with strongest expected relationship.

*p < 0.05; **p < 0.01; ***p < 0.001.

*Abbreviations:* *AZ* AstraZeneca, *BCSS* Breathlessness, Cough and Sputum Scale, *E-RS* Exacerbations of Chronic Pulmonary Disease Tool – Respiratory Symptoms, *FEV*_*1*_ forced expiratory volume in 1 second, *mMRC* modified Medical Research Council dyspnoea scale.

**Table 4:** **Correlations** ^**†**^ **between E-RS scores and indicators of health status, exercise capacity, and functional performance**

	**Mpex** ^**†**^		**AZ 1** ^**†**^			**AZ 2** ^**†**^	
	**SGRQ total** ^**‡**^ **(N = 233)**	**6MWT distance** ^**‡**^ **(N = 231)**	**SGRQ-C total** ^**‡**^ **(N = 745)**	**6MWT distance** ^**‡**^ **(N = 745)**	**FPI-SF total** ^**‡**^ **(N = 155)**	**SGRQ-C total** ^**‡**^ **(N = 586)**	**ISWT distance** ^**‡**^ **(N = 596)**
E-RS Scores							
RS-Total Score	0.65**	−0.30***	0.52**	−0.14***	−0.43***	0.51**	−0.18***
RS-Breathlessness	0.60**	−0.33***	0.54**	−0.15***	−0.47***	0.52**	−0.19***
RS-Cough and Sputum	0.45**	−0.14*	0.42**	−0.10**	−0.28***	0.41**	−0.12**
RS-Chest Symptoms	0.50**	−0.17**	0.40**	−0.10**	−0.28***	0.44**	−0.15***

^**†**^Spearman's rank-order correlation; E-RS mean weekly scores (Day −7 to Day −1).

^‡^Clinic visit, Day 1, prior to treatment.

**Bold** coefficients identify constructs with strongest expected relationship.

*p < 0.05; **p < 0.01; ***p < 0.001.

*Abbreviations:* *6MWT* 6-minute walk test, *AZ* AstraZeneca, *E-RS* Exacerbations of Chronic Pulmonary Disease Tool – Respiratory Symptoms, *FPI-SF* Functional Performance Inventory – Short Form, *ISWT* Incremental Shuttle Walk Test, *SGRQ* St. George’s Respiratory Questionnaire, *SGRQ-C* St. George’s Respiratory Questionnaire for COPD.

**Figure 1:**
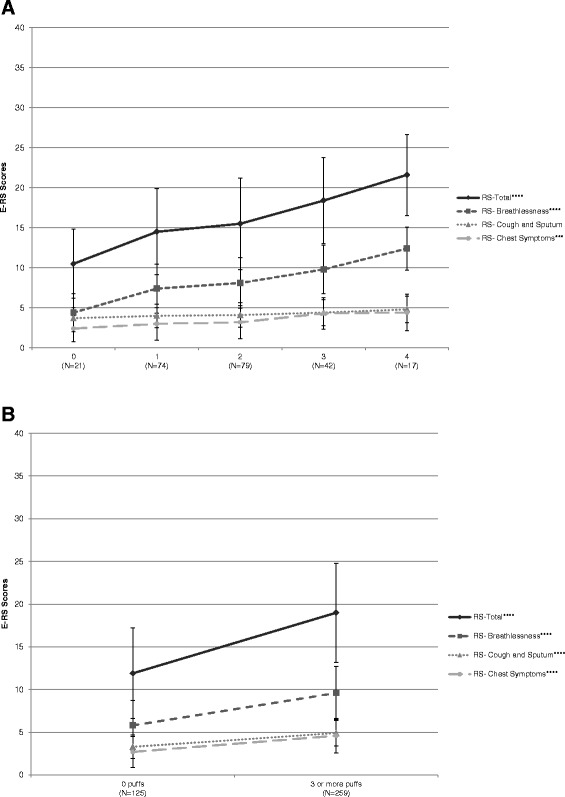
**E-RS Scores by Level of Dyspnoea and Rescue Medication Use. A**: Level of Dyspnea (mMRC^†^). ^†^Mpex data only; E-RS mean weekly scores (Day −7 to Day −1); ANCOVA controlling for age, co-morbidity, and baseline FEV_1_. ***p < 0.001; ****p < 0.0001 from global test of differences between levels. **Abbreviations:** ANCOVA = analysis of covariance; E-RS = Exacerbations of Chronic Pulmonary Disease Tool – Respiratory Symptoms; FEV_1_ = forced expiratory volume in 1 second; mMRC = modified Medical Research Council dyspnoea scale. **B**: Rescue Medication Use^**†**^. ^†^Results for AZ 1 data shown; similar results were found in AZ 2 data; E-RS and rescue medication use are mean weekly values (Day −7 to Day −1); Students *t*-test. ****p < 0.0001. **Abbreviations:** AZ = AstraZeneca; E-RS = Exacerbations of Chronic Pulmonary Disease Tool – Respiratory Symptoms.

Known-groups validity, evaluating E-RS scores by mMRC dyspnoea level (Mpex) and rescue medication use (AZ 1 and 2) at baseline, is shown in Figure 1. As expected, the strongest relationship was with RS-Total and RS-Breathlessness scores.

### Responsiveness

**Figure 2:**
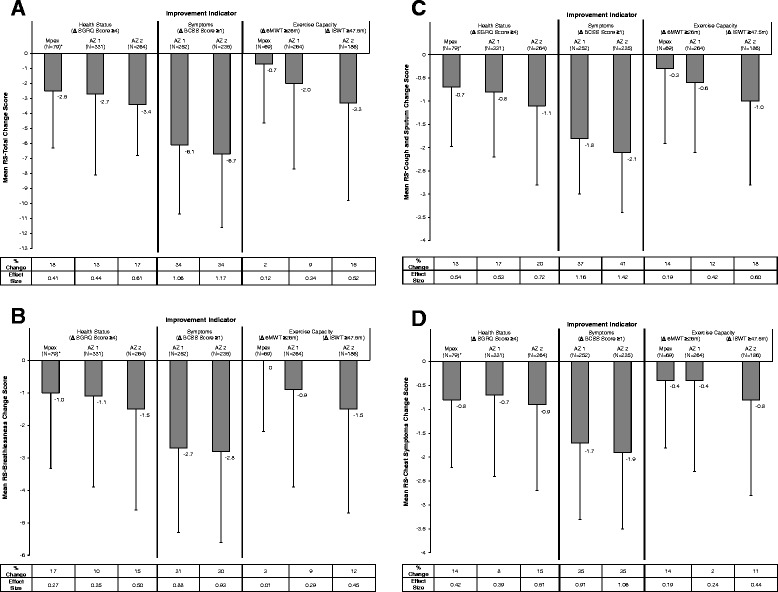
**E-RS Score Improvements by Health Status, Respiratory Symptom, or Exercise Capacity Improvement at 3 Months. A**: RS-Total. *Data from one subject with an extreme positive (worse) E-RS Cough & Sputum score (8 SD) removed from the analysis. **Abbreviations:** 6MWT = 6-minute walk test; AZ = AstraZeneca; BCSS = Breathlessness, Cough and Sputum Scale; ISWT = Incremental Shuttle Walk Test; SGRQ = St. George’s Respiratory Questionnaire. **B**: RS-Breathlessness. *Data from one subject with an extreme positive (worse) E-RS Cough & Sputum score (8 SD) removed from the analysis. **Abbreviations:** 6MWT = 6-minute walk test; AZ = AstraZeneca; BCSS = Breathlessness, Cough and Sputum Scale; ISWT = Incremental Shuttle Walk Test; SGRQ = St. George’s Respiratory Questionnaire. **C**: RS-Cough and Sputum. *Data from one subject with an extreme positive (worse) E-RS Cough & Sputum score (8 SD) removed from the analysis. **Abbreviations:** 6MWT = 6-minute walk test; AZ = AstraZeneca; BCSS = Breathlessness, Cough and Sputum Scale; ISWT = Incremental Shuttle Walk Test; SGRQ = St. George’s Respiratory Questionnaire. **D**: RS-Chest Symptoms. *Data from one subject with an extreme positive (worse) E-RS Cough & Sputum score (8 SD) removed from the analysis. **Abbreviations:** 6MWT = 6-minute walk test; AZ = AstraZeneca; BCSS = Breathlessness, Cough and Sputum Scale; ISWT = Incremental Shuttle Walk Test; SGRQ = St. George’s Respiratory Questionnaire.

Figure 2 displays E-RS score changes by improvement indicator and trial. For patients whose health status improved from baseline to 3 months, RS-Total scores declined (i.e., improved) by an average of −2.5 to −3.4 on the 40 point scale, corresponding to 13% to 18% symptomatic change (ES = 0.41 to 0.61). Mean improvements in RS-Total score corresponding to BCSS changes ≥1 [24] exceeded −6 points (34%, ES > −1.0). Symptomatic improvement in patients showing improvements in exercise capacity ranged from −0.6 (2% change, ES 0.12) to −3.3 points (15% change, ES 0.52). E-RS subscales (Figure 2B to 2D) showed similar patterns within and across indicators. Responder estimates using distribution-based methods are shown in Table 5. Results of exploratory analyses examining E-RS score changes in subjects experiencing health status deterioration from baseline to week 12 are shown in Additional file 1: Tables E5 and E6.

**Table 5:** **E-RS Responder estimates using distribution-based methods by trial: ½ SD and SEM** ^*^

**Scale Method** ^**†**^	**Mpex**	**AZ 1**	**AZ 2**
	**(N = 235)**	**(N = 749)**	**(N = 597)**
RS-Total			
½ SD	2.97	3.00	2.995
SEM	1.88	1.47	1.34
RS-Breathlessness			
½ SD	2.97	1.59	1.56
SEM	1.57	0.78	0.76
RS-Cough and Sputum			
½ SD	1.04	0.78	0.80
SEM	1.44	0.81	0.75
RS-Chest Symptoms			
½ SD	1.04	0.97	0.96
SEM	0.42	0.47	0.51

*$$ SEM=SD\sqrt{1-\alpha } $$.

^†^Weekly scores at baseline, Day −7 to Day 1; Ranges: RS-Total: 0 to 40; RS-Breathlessness: 0 to 17; RS-Cough and Sputum: 0 to 11; RS-Chest Symptoms: 0 to 12; higher scores = more severe symptoms.

*Abbreviations:* *AZ* AstraZeneca, *E-RS* Exacerbations of Chronic Pulmonary Disease Tool – Respiratory Symptoms, *SD* standard deviation, *SEM* standard error of measurement.

## Discussion

Determining the extent to which interventions provide respiratory symptom relief requires randomized trials with precise endpoint measurement. Comparing treatment effects across studies, e.g., meta-analysis, requires comparable metrics. To date, there has been no standardized, reliable, and valid diary for evaluating the cardinal respiratory symptoms of COPD developed with regulatory standards for drug development tools in mind [17-19]. This paper describes the performance of the E-RS in 3 independent, international RCTs of stable, symptomatic patients with moderate to severe airway obstruction receiving maintenance therapy.

RS-Total and subscale scores exhibited evidence of reliability, validity, and responsiveness in each trial. Reliability was estimated using tests of internal consistency, a measure of scale coherence, and reproducibility over time. Across studies, estimates were strong, exceeding the 0.70 target for use in clinical trials [32,35] and the more conservative 0.80 standard [33], suggesting E-RS scores are precise, with relatively low levels of measurement error. With one exception, values were similar to those reported previously [20]. The exception was the internal consistency estimates for the RS-Cough and Sputum in the Mpex sample (ICC for daily measurements 0.40; 0.52 weekly). This provides an interesting case study for reliability estimation. It is well known, and a function of the formula for coefficient alpha, that reliability is a characteristic of the scale scores in a study population. An inclusion criterion for the Mpex trial was mucopurulent sputum on most days. During baseline and over the study period, 6%–11% of this patient sample reported that it was extremely difficult to bring up mucus (phlegm); for comparison purposes, the ceiling effect for this item was observed in less than 2% of the other 2 samples. Ceiling effects in one variable will attenuate correlations that include this variable and hence reduce reliability coefficients. Thus, the lower coefficient for internal consistency in the RS-Cough and Sputum scale in the Mpex study sample is consistent the sputum severity characteristics of this sample. A drug that eased difficulty coughing up sputum should lead to a downward shift (improvement) in this aspect of cough and sputum, reducing the proportion of patients with extreme difficulties. The subscale should also show higher reliability levels with effective treatment, as the ceiling effect for this item is reduced or eliminated and equilibrated across the items comprising the scale.

The 2-day test-retest estimates in the Mpex data indicate consecutive daily scores are reproducible in patients who report no change in their lung condition over 2 days. The lower estimates observed over a 6-day interval in all subjects during the pre-randomization baseline run-in suggest some degree of variability in patients assumed to be stable over this period. Similar patterns were observed in the initial testing of E-RS scores [20]. Together, these results indicate a diary capturing symptom severity each day and averaged over time would be more accurate than periodic symptom assessments with longer recall periods commonly used in health status questionnaires, such as the SGRQ or Chronic Respiratory Questionnaire (CRQ) [36]. This not only enhances the precision of symptom severity estimates and treatment effects, but permits study of day-to-day symptom variability, an area in need of further research.

The magnitude and pattern of correlations and known-group differences were consistent with what would be expected of a valid patient-reported measure of respiratory symptoms. Coefficients were highest in tests of concurrent validity, i.e., between E-RS and BCSS scores, since both measure respiratory symptoms. In tests of convergent validity, E-RS and SGRQ health status scores were also strongly and consistently correlated across the 3 trials, although somewhat weaker than in the initial development study (0.75, 0.69, 0.58, 0.52 for RS-Total and subscale scores, respectively [20]). Another widely used measure of health status, the COPD Assessment Test (CAT) [37] was not administered in these trials; given the strong relationship between the SGRQ and CAT (e.g., r = 0.69 to 0.87) [38], one would expect the E-RS and CAT to be highly correlated as well. The E-RS should be complementary to heath status questionnaires such as the SGRQ, CAT, and CRQ in clinical trials since it captures day-to-day severity and variability of the cardinal respiratory symptoms of COPD with minimal recall bias and with content, subscale structure, and scores that capture these symptoms. To optimize data quality, particularly over lengthy trials, the E-RS should be completed as part of the EXACT, on a pretested, user-friendly electronic device programmed with reminders; subjects should be trained on the device and monitored for compliance during the course of the study [39].

Patients with more severe symptoms reported poorer functional performance, as measured by the FPI-SF. In keeping with divergent validity, correlations between respiratory symptoms and airway obstruction (FEV_1_% predicted) were weak, although statistically significant. Of the 3 subscales, RS-Breathlessness was consistently the strongest correlate of FEV_1_% predicted, mMRC, rescue medication use, and functional performance, all indicators or effects of dyspnoea. This is consistent with convergent validity for RS-Breathlessness and divergent validity for RS-Cough and Sputum and RS-Chest Symptoms.

E-RS scores were sensitive to change in patients showing improvement in health status (3 of 3 studies), symptoms (2 of 2 studies) or exercise tolerance (3 of 3 studies) from baseline to month 3. Exploratory analyses suggest E-RS scores are also sensitive to symptomatic worsening over 12 weeks.

In the initial testing of the E-RS, only distribution-based methods (½ standard deviation of baseline values) were available for responder definitions of symptomatic improvement; these gave values of: RS-Total: 3.35; RS-Breathlessness: 1.85; RS-Cough and Sputum: 1.15; and RS-Chest Symptoms: 1.05 [20]. Such methods are largely unvalidated in terms of their relationship to the patient’s actual experience and may give values higher than the “true” responder threshold [40]. The current set of analyses support this caveat; the initial estimates should be interpreted as moderate to large, and not as minimal. Based on results from these 3 trials, across criterion-variable and distribution-based methods, variable and distribution-based methods, the following responder definitions for symptomatic improvement are proposed:

- RS-Total ≥ −2.0 (scale range: 0–40)
- RS-Breathlessness ≥ −1.0 (scale range: 0–17)
- RS-Cough and Sputum ≥ −0.70 (scale range 0–11)
- RS-Chest Symptoms ≥ −0.70 (scale range: 0–12)

Descriptive statistics and figures showing E-RS change scores over 12 weeks for responders and non-responders are provided in Additional file 1: Table E4 and Figures E1a–E1d. Results of the exploratory analyses suggest symmetric thresholds for symptomatic improvement and decline (i.e., applying ≥ + 2.0; ≥ + 1.0; ≥ + 0.70; ≥ + 0.70 for Total and subscale scores, respectively for symptom worsening) (see Additional file 1: Tables E5 and E6). Given the magnitude of symptomatic improvement in responders and symptomatic decline in those whose symptoms worsened, these definitions may also be conservative estimates. Further research is needed in studies with global ratings of change, compounds showing treatment effects in several efficacy endpoints, including respiratory symptoms, and longer studies in patients at risk of decline.

Although the results presented here provide evidence that E-RS scores are reliable, valid, and sensitive to change in international RCT settings, several limitations should be noted. First, because the experimental drugs showed no therapeutic effects, responsiveness to treatment relative to placebo could not be evaluated. Tests of sensitivity to change and responder definitions were based on changes observed in criterion variables generally associated with symptomatic change. Results reported by Beier et al. [41] indicate E-RS scores are sensitive to symptomatic improvement with effective treatment over 6 weeks. In that trial, significant within- and between- group treatment effects were observed for aclidinium bromide and tiotropium versus placebo (E-RS-Total, RS-Breathlessness, and RS Chest Symptoms; significant effects for RS-Cough and Sputum for aclidinium only).

A second limitation is related to the study samples. Each trial enrolled symptomatic and clinically stable patients, consistent with the target population for the measure. In addition, the inclusion criteria specified a history of 1 or more clinic visits or hospitalizations for COPD exacerbation the prior 12 months. Although it is reasonable to expect the E-RS to perform similarly in patients without this history, further study should be undertaken to test this assumption. Finally, these trials were limited to 3 to 6 months; longer studies would permit an evaluation of the patterns and persistence of symptomatic improvement and worsening, including further study of thresholds for meaningful improvement and deterioration.

## Conclusion

The E-RS provides a reliable, valid, and responsive method for quantifying respiratory symptom severity in clinical studies of COPD. Because the 11-item E-RS is embedded in the 14-item EXACT, a single diary can be used to evaluate the effects of treatment on day-to-day symptom severity in stable disease, using E-RS scores, and on acute exacerbations of COPD using the EXACT scoring algorithms.

## Additional file

Additional file 1:
**Showing E-RS score reproducibility (test-retest) results; one table and one set of figures showing E-RS scores over time in responders and non-responders; two tables and a description of symptomatic change in subjects (non-responders) with indication of worsening; and a detailed description of the E-RS instrument.**
