# Supplementary material for: Measuring respiratory symptoms of COPD: performance of the EXACT- Respiratory Symptoms Tool (E-RS) in three clinical trials
Source: Respir Res. 2014 Oct 7;15(1):124. doi: 10.1186/s12931-014-0124-z (PMC4203869; doi:10.1186/s12931-014-0124-z)
Supplement: Additional file 1: — Showing E-RS score reproducibility (test-retest) results; one table and one set of figures showing E-RS scores over time in responders and non-responders; two tables and a description of symptomatic change in subjects (non-responders) with indication of worsening; and a detailed description of the E-RS instrument. [file 12931_2014_124_MOESM1_ESM.pdf]

Measuring Respiratory Symptoms of COPD:
Performance of the EXACT- Respiratory Symptoms Tool (E-RS) in Three Clinical Trials

NK Leidy, PhD[1§]; LT Murray, MPH[1]; BU Monz, MD, MA, MPH[2]; L Nelsen, MHS[3];
M Goldman, MD[4]; PW Jones, PhD, FRCP[5]; EJ Dansie, PhD[1]; S Sethi, MD[6]

[1]Evidera, Bethesda, MD, US; [2]Formerly Boehringer Ingelheim GmbH, Ingelheim, Germany;
[3]Merck, Sharp & Dohme, Corp, Whitehouse Station, NJ; [4]AstraZeneca, Wilmington, DE, US;
[5]St. George's, University of London, London, UK; [6]University of Buffalo, Buffalo, NY, US

ADDITIONAL FILE 1 - ONLINE SUPPLEMENT

E-RS Score Reliability

Data Source: Mpex

Table E1 - Seven-day Reproducibility of E-RS Daily Scores during Study Run-in Period* (N=235)

E-RS Scores	N†	Day -7 Mean (SD)	Day -1 Mean (SD)	Mean Difference‡ (SD)	p Value‡	Effect Size§	ICC¶
RS-Total Score	168	15.7 (6.87)	16.4 (6.33)	-0.8 (4.98)	0.0508	-0.12	0.71
RS-Breathlessness Score	168	8.3 (3.93)	8.6 (3.77)	-0.4 (2.80)	0.1055	-0.09	0.73
RS-Cough and Sputum Score	168	4.2 (1.96)	4.3 (1.69)	0.0 (1.68)	0.7486	-0.02	0.58
RS-Chest Symptoms Score	168	3.2 (2.36)	3.5 (2.22)	-0.4 (1.79)	0.0093	-0.16	0.69

*Pre-treatment, Cycle 1, Day -7; Day -1. Baseline scores represent the participant's stable or usual state.
†Number of study participants.
‡Mean difference = average Day -7 RS-score - average Day -1 RS score; p value from paired *t*-test.
§Effect size = Day -7 RS score - Day -1 RS score/SD of Day -1 RS score.
¶Intraclass correlation coefficient (ICC) based on random-effect model.

Table E2 - Reproducibility of E-RS Scores in Patients Reporting No Change* for 2 Consecutive Days before Treatment (Cycle 1, Day -2 and Day -1) (N=235)

E-RS Scores	N†	Day -2 Mean (SD)	Day -1 Mean (SD)	Mean Difference‡ (SD)	p Value‡	Effect Size§	ICC¶
RS-Total Score	170	15.0 (5.94)	15.3 (5.67)	-0.3 (2.44)	0.0755	-0.06	0.91
RS-Breathlessness Score	170	7.8 (3.52)	8.1 (3.32)	-0.3 (1.71)	0.0231	-0.09	0.87
RS-Cough and Sputum Score	170	4.0 (1.70)	4.0 (1.73)	0.0 (0.97)	0.8749	-0.01	0.84
RS-Chest Symptoms Score	170	3.2 (2.26)	3.2 (2.12)	0.0 (1.03)	0.7666	-0.01	0.89

*Patients reporting "about the same" on item "Overall, how was your lung condition today compared to yesterday?"
†Number of study participants.
‡Mean difference = average Day -2 RS-score - average Day -1 RS score; p value from paired *t*-test. Where data are missing for one of those days, the most recent pair of consecutive days was used (i.e., Day -2 and Day -3).
§Effect size = Day -2 RS score - Day -1 RS score/SD of Day -1 RS score.
¶Intraclass correlation coefficient (ICC) based on random-effect model.

Table E3 - Reproducibility of E-RS Mean Weekly Scores from Cycle 1, Day -7 to Day -1 to Cycle 3, Day 22 to Day 28 in Unchanged Patients Identified Using the SGRQ* (N=235)

E-RS Scores	N†	Cycle 3, Day 22 to Day 28 Mean (SD)	Cycle 1, Day -7 to Day -1 Mean (SD)	Mean Difference‡ (SD)	p Value‡	Effect Size§	ICC¶
RS-Total Score	63	15.5 (5.16)	15.8 (5.77)	-0.3 (4.26)	0.6160	-0.05	0.70
RS-Breathlessness Score	63	8.6 (3.18)	8.5 (3.40)	0.0 (2.30)	0.9173	0.01	0.76
RS-Cough and Sputum Score	63	4.0 (1.40)	4.1 (1.59)	-0.1 (1.45)	0.4640	-0.09	0.53
RS-Chest Symptoms Score	63	2.9 (1.97)	3.1 (2.08)	-0.2 (1.63)	0.4240	-0.08	0.68

*For SGRQ total scores, 'unchanged' defined as a score change greater than -4.00 and less than 4 from baseline (Cycle 1, Day 1) to Cycle 4, Day 1.
†Number of study participants.
‡Mean difference = average Day -2 RS-score - average Day -1 RS score; p value from paired *t*-test.
§Effect size = Cycle 3, Day 22 to Day 28 RS score-Cycle 1, Day -7 to Day -1/SD of Cycle 1, Day -7 to Day -1 RS score.
¶Intraclass correlation coefficient (ICC) based on random-effect model.

Mean change (SD) for patients with no change in health status (SGRQ) from baseline to Day 28 was -0.3 (4.26) for the RS-Total and ranged from -0.2 (1.63) to 0.0 (2.30) for the domain scores. Mean percent change for the RS-Total score was 4.1% with an ES of -0.05. Domain mean percent changes ranged from 0.2% to 14.1% with ES ranging from -0.09 to 0.01.

E-RS Score Responders and Non-Responders

Descriptive statistics for E-RS scores using the proposed responder definitions for symptomatic improvement are shown in Table E4.

Table E4 - Change in E-RS Scores Baseline to Week 12 by Responder Status[*] and Trial

	N	Baseline Mean (SD)	Week 12 Mean (SD)	Change Mean (SD)	Effect Size	% Change Mean (SD)
**RS-Total (Range: 0–40)**						
Responders (≥ -2 points)						
Mpex	84	17.6 (6.0)	12.4 (6.0)	-5.3 (3.0)	0.88	-33 (19)
AZ 1	284	17.6 (6.0)	11.5 (5.5)	-6.2 (4.1)	1.02	-36 (19)
AZ 2	255	20.0 (5.6)	13.0 (5.8)	-6.9 (4.4)	1.24	-36 (20)
Non-Responders (< -2 points)						
Mpex	137	14.4 (5.6)	16.3 (6.3)	1.9 (3.3)	0.34	16 (33)
AZ 1	438	14.8 (5.7)	16.8 (6.5)	2.0 (3.7)	0.35	18 (43)
AZ 2	322	16.8 (6.0)	18.5 (6.6)	1.7 (2.9)	0.28	12 (24)
**RS-Breathlessness (Range: 0–17)**						
Responders (≥ -1 point)						
Mpex	72	8.3 (3.9)	5.4 (3.7)	-2.9 (1.7)	0.74	-43 (28)
AZ 1	266	8.6 (3.1)	5.5 (2.9)	-3.2 (2.0)	1.02	-39 (23)
AZ 2	244	9.5 (2.9)	6.2 (3.0)	-3.3 (2.2)	1.17	-36 (22)
Non-Responders (< -1 point)						
Mpex	149	8.1 (3.3)	9.3 (3.3)	1.2 (1.9)	0.37	21 (45)
AZ 1	456	7.5 (3.1)	8.7 (3.4)	1.1 (1.9)	0.36	23 (54)
AZ 2	333	8.5 (2.3)	9.5 (3.5)	1.0 (1.6)	0.30	16 (41)
**RS-Cough and Sputum (Range: 0–11)**						
Responders (≥ -0.7 point)						
Mpex	85	4.6 (1.3)	2.9 (1.3)	-1.7 (1.0)	1.39	-39 (22)
AZ 1	309	4.7 (1.5)	3.0 (1.4)	-1.8 (1.0)	1.18	-39 (21)
AZ 2	267	5.2 (1.6)	3.1 (1.6)	-2.1 (1.2)	1.31	-41 (23)
Non-Responders (< -0.7 point)						
Mpex	136	3.8 (1.7)	4.4 (1.6)	0.5 (1.1)	0.31	30 (79)
AZ 1	413	3.8 (1.5)	4.2 (1.6)	0.4 (1.0)	0.29	16 (42)
AZ 2	310	4.3 (1.5)	4.6 (1.7)	0.4 (0.8)	0.25	12 (30)
**RS-Chest Symptoms (Range: 0–12)**						
Responders (≥ -0.7 point)						
Mpex	78	4.1 (2.0)	2.1 (1.9)	-2.0 (1.1)	1.00	-58 (29)
AZ 1	270	4.5 (1.8)	2.5 (1.7)	-2.0 (1.3)	1.08	-48 (26)
AZ 2	240	5.2 (1.7)	3.1 (1.7)	-2.2 (1.3)	1.30	-43 (24)
Non-Responders (< -0.7 point)						
Mpex	143	2.9 (2.0)	3.5 (2.2)	0.6 (1.1)	0.28	27 (95)
AZ 1	452	3.4 (1.9)	4.1 (2.2)	0.7 (1.2)	0.39	43 (175)
AZ 2	337	4.2 (2.0)	4.8 (2.2)	0.6 (1.0)	0.31	19 (44)

[*]Responder: Symptomatic improvement; Non-Responder: No change or worsening.

Weekly mean changes in E-RS symptom scores over 12 weeks by responder status are shown in Figures E1a–E1d.

Figure E1a - Mean (SE) Change in RS-Total Scores Over 12 Weeks by Responder Status* and Trial

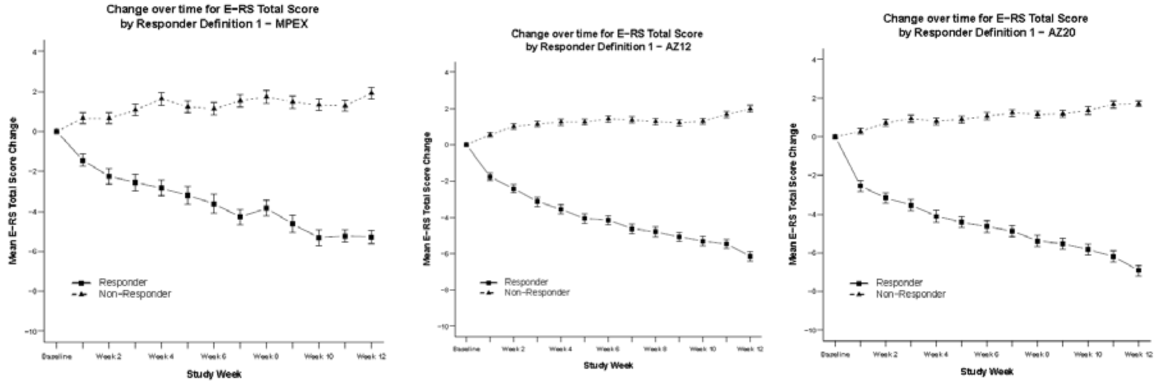

Figure E1b - Mean (SE) Change in RS-Breathlessness Scores Over 12 Weeks by Trial

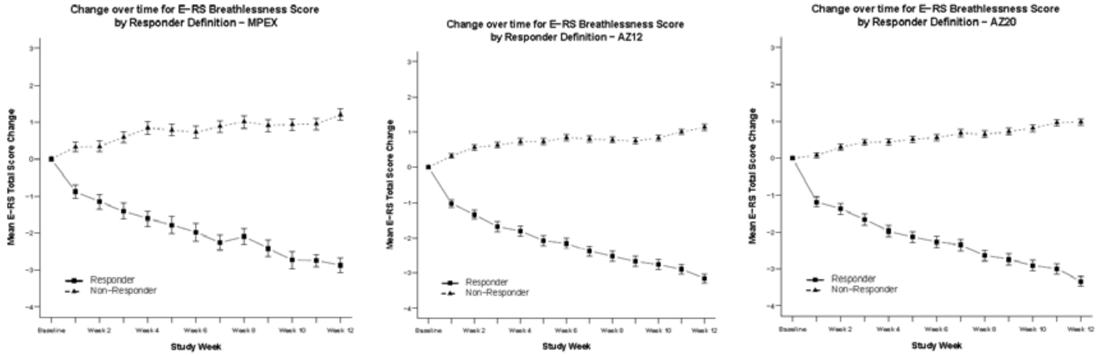

Figure E1c - Mean (SE) Change in RS-Cough and Sputum Scores Over 12 Weeks by Trial

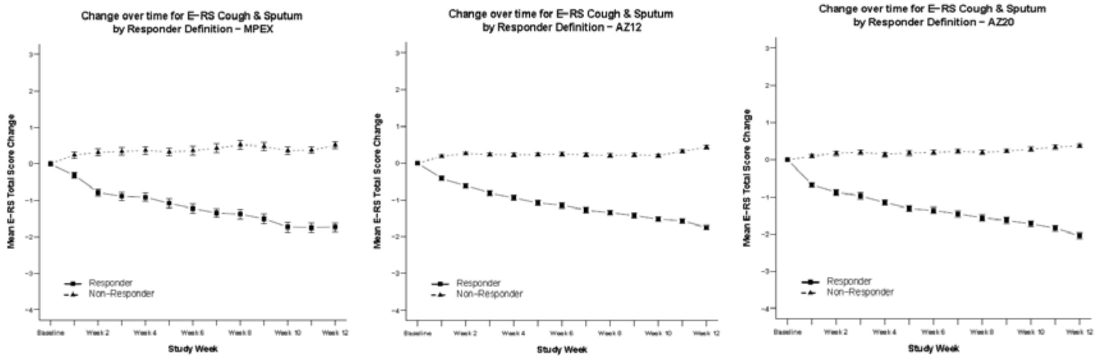

Figure E1d - Mean (SE) Change in RS-Chest Symptoms Scores Over 12 Weeks by Trial

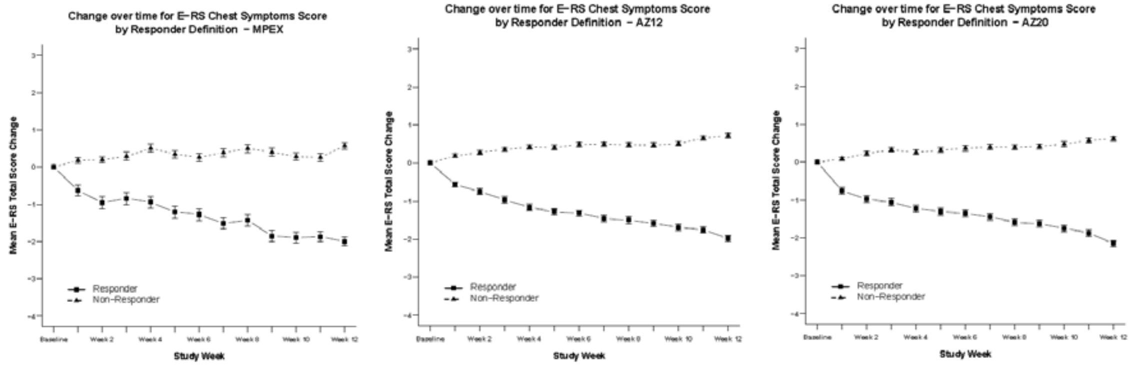

*Responder: Symptomatic improvement; Non-Responder: No change or worsening. Higher E-RS Scores: More severe symptoms; Negative Change Scores: Symptomatic improvement

E-RS Scores for Non-Responders with Indications of Worsening

Exploratory analyses were performed to examine E-RS score changes in subjects experiencing deterioration in health status (SGRQ ≥4 points) or on an alternative measure of symptoms (BCSS ≥1 point) over 12 weeks. Student's *t*-test, percent change, and ES were used to evaluate the magnitude of E-RS score change from baseline. Results are shown in Table E5.

Table E5 - E-RS Change Score Parameters for Subjects Reporting Worsening Condition Baseline† to Week 12

Criterion<br>Trial (n)<br>Parameter	RS-Total	RS-Breathlessness	RS-Cough and Sputum	RS-Chest Symptoms
**SGRQ ≥ 4.0**				
**Mpex (n=39)**				
Mean (SD)	2.0 (4.61)**	1.4 (2.28)***	0.10 (1.58)	0.50 (1.66)
% Change	19	24	14	18
Effect Size	0.38	0.3	0.09	0.21
**AZ 1 (n=185)**				
Mean (SD)	0.9 (5.9)*	0.5 (2.9)*	0.0 (1.7)	0.4 (1.9)**
% Change	10	12	8	29
Effect Size	0.16	0.16	0.02	0.22
**AZ 2 (n=124)**				
Mean (SD)	-0.4 (5.9)	0.0 (2.8)	-0.3 (1.5)**	-0.1 (1.7)
% Change	2	6	5	3
Effect Size	0.07	0.01	0.21	0.03
**BCSS ≥ 1.0**				
**AZ 1 (n=241)**				
Mean (SD)	3.4 (4.4)****	1.5 (2.4)****	0.8 (1.2)****	1.0 (1.6)****
% Change	31	31	30	68
Effect Size	0.56	0.49	0.54	0.54
**AZ 2 (n=61)**				
Mean (SD)	5.1 (3.4)*	2.2 (2.2)*	1.4 (1.0)*	1.5 (1.4)*
% Change	34	33	40	47
Effect Size	0.89	0.73	1.01	0.70

†Deterioration in health status (SGRQ) or symptoms (BCSS).

*p<0.05; **p<0.01; ***p<0.001; ****p<0.0001.

To evaluate the possibility of symmetric thresholds (same absolute value applied to improvement and worsening), the following threshold values were used to identify patients with symptomatic deterioration (≥2.0, ≥1.0, ≥0.70, and ≥0.70 for the total and 3 subscales, respectively). Table E6 shows the mean and percentage change in E-RS score and ES estimates for these patients.

Table E6 - E-RS Change Scores Baseline to Week 12 for Subjects Exhibiting Symptomatic Decline*

E-RS Scale<br>Trial	N	Baseline<br>Mean (SD)	Week 12<br>Mean (SD)	Change<br>Mean (SD)	Effect<br>Size	% Change<br>Mean (SD)
**RS-Total (Range: 0–40)**						
Symptomatic Decline (≥ 2.0 points)						
Mpex	55	13.8 (4.82)	18.8 (5.59)	4.9 (3.15)	1.02	42.0 (36.98)
AZ 1	158	14.7 (5.87)	20.3 (6.54)	5.6 (3.96)	0.96	49.9 (58.13)
AZ 2	118	16.2 (5.79)	20.9 (6.30)	4.7 (2.58)	0.81	34.5 (25.48)
**RS-Breathlessness (Range: 0–17)**						
Worse Breathlessness (≥ 1.0 points)						
Mpex	61	7.5 (2.96)	10.5 (2.71)	2.9 (1.81)	1.00	53.6 (53.26)
AZ 1	192	7.3 (3.01)	10.0 (3.29)	2.7 (1.88)	0.90	51.9 (71.47)
AZ 2	137	8.1 (3.12)	10.6 (3.34)	2.5 (1.48)	0.79	41.0 (49.80)
**RS-Cough and Sputum (Range: 0–11)**						
Worse Cough & Sputum (≥ 0.7 points)						
Mpex	42	3.0 (1.62)	4.8 (1.68)	1.8 (1.02)	1.14	97.8 (114.9)
AZ 1	114	3.5 (1.47)	5.2 (1.71)	1.7 (1.12)	1.14	59.3 (56.71)
AZ 2	90	4.0 (1.35)	5.5 (1.49)	1.4 (0.68)	1.08	41.9 (34.30)
**RS-Chest Symptoms (Range: 0–12)**						
Worse Chest Symptoms (≥ 0.7 points)						
Mpex	46	2.5 (1.70)	4.4 (1.94)	1.8 (1.03)	1.08	103.4 (130.9)
AZ 1	173	3.3 (1.87)	5.2 (2.16)	1.9 (1.21)	1.03	121.1 (262.9)
AZ 2	126	4.0 (1.88)	5.7 (2.07)	1.7 (0.90)	0.89	55.4 (49.63)

*Using E-RS threshold estimates for decline

These results suggest the E-RS score thresholds for deterioration may be smaller than those used to identify treatment responders showing symptomatic improvement. Further study of the relationship between symptomatic deterioration of various magnitudes and other clinical outcomes, including exacerbations and mortality, over longer study periods is warranted.

Table E7 - Content and Structure of the EXACT and EXACT-RS[a]

Scale	Item-level Construct[1]	Item Number
**RS-Breathlessness**		
	Breathless today[2]	7
	Breathless with activity[3]	8
	Short of breath - personal care[3]	9
	Short of breath - indoor activity[3]	10
	Short of breath - outdoor activity[3]	11
**RS-Cough and Sputum**		
	Cough frequency[3]	2
	Mucus quantity[3]	3
	Difficulty with mucus[2]	4
**RS-Chest Symptoms**		
	Congestion[2]	1
	Discomfort[2]	5
	Tightness[2]	6
**Respiratory Symptoms**	**E-RS Total**	**11 items**
**Additional Attributes**		
Used in the EXACT total score for characterizing exacerbations, specifically.	Tired or weak[2]	12
	Sleep disturbance[2]	13
	Scared or worried[2]	14
**EXACT – Exacerbations**	**EXACT Total Score**	**14 items**

[a] All 14 items of the EXACT are administered as a daily diary; E-RS scoring uses only the respiratory symptom items, with scoring based on summation to yield ordinal-level scales.

1. Items are worded as simple questions. Recall: "Today". Patients are asked to complete the diary every evening before they go to bed, reflecting on their symptoms that day. The formatted instrument is available through exactpro@evidera.com.

2. 5-point scale, Not at all to Extremely

3. 5-point scale – other
